# Thiocyanate Reduces Motor Impairment in the hMPO-A53T PD Mouse Model While Reducing MPO-Oxidation of Alpha Synuclein in Enlarged LYVE1/AQP4 Positive Periventricular Glymphatic Vessels

**DOI:** 10.3390/antiox11122342

**Published:** 2022-11-26

**Authors:** Wanda F. Reynolds, Ernst Malle, Richard A. Maki

**Affiliations:** 1 Sanford Burnham Prebys Medical Discovery Institute, La Jolla, CA 92037, USA; 2 Gottfried Schatz Research Center, Division of Molecular Biology and Biochemistry, Medical University of Graz, 8010 Graz, Austria

**Keywords:** alpha synuclein, aquaporin 4, carbamylation, endothelial hyaluronan receptor 1, glial fibrillary acidic protein, glymphatics, hypochlorous acid, myeloperoxidase, nitration, Parkinson’s disease, reactive oxygen species

## Abstract

Parkinson’s disease (PD) is due to the oxidation of alpha synuclein (αSyn) contributing to motor impairment. We developed a transgenic mouse model of PD that overexpresses the mutated human αSyn gene (A53T) crossed to a mouse expressing the human MPO gene. This model exhibits increased oxidation and chlorination of αSyn leading to greater motor impairment. In the current study, the hMPO-A53T mice were treated with thiocyanate (SCN^−^) which is a favored substrate of MPO as compared to chlorine. We show that hMPO-A53T mice treated with SCN^−^ have less chlorination in the brain and show an improvement in motor skills compared to the nontreated hMPO-A53T mice. Interestingly, in the hMPO-A53T mice we found a possible link between MPO-related disease and the glymphatic system which clears waste including αSyn from the brain. The untreated hMPO-A53T mice exhibited an increase in the size of periventricular glymphatic vessels expressing the glymphatic marker LYVE1 and aquaporin 4 (AQP4). These vessels also exhibited an increase in MPO and HOCl-modified epitopes in the glymphatic vessels correlating with loss of ependymal cells lining the ventricles. These findings suggest that MPO may significantly promote the impairment of the glymphatic waste removal system thus contributing to neurodegeneration in PD. Moreover, the inhibition of MPO chlorination/oxidation by SCN^−^ may provide a potential therapeutic approach to this disease.

## 1. Introduction

Parkinson’s disease (PD), the second most common neurodegenerative disease, is associated with the selective loss of dopaminergic neurons in the substantia nigra pars compacta leading to tremor, bradykinesia, rigidity, and postural instability (reviewed [1,2]). αSyn is considered to be a major contributor to the development of PD due to its susceptibility to oxidation leading to formation of aggregates [3,4]. While there are several pathways that can lead to the oxidation of αSyn, reactive oxygen species (ROS) promoting halogenation and nitration are likely to be involved [5,6,7,8,9,10].

Myeloperoxidase (MPO), abundantly present in neutrophils (up to 5% of cellular proteins) and monocytes (up to 1% of cellular protein) is commonly known to generate ROS and reactive nitrogen species [11]. MPO is a component of the armamentarium of the innate immune system, present at high levels in storage vesicles in phagocytes [12]. When neutrophils and monocytes engulf microbes, MPO-containing vesicles fuse with the phagosome releasing the enzyme which reacts in the presence of hydrogen peroxide (H_2_O_2_) with chloride ions (Cl^−^) to produce the potent oxidant, hypochlorous acid (HOCl, commonly known as bleach) [13,14]. MPO can also react with other halides (Br^−^ and I^−^) and pseudohalide ions (thiocyanate, SCN^−^) to form hypohalous acids such as hypobromous acid (HOBr), hypoiodus acid (HOI), and hypothiocyanous acid (HOSCN), respectively [15,16]. Nitration is also an oxidation product by the MPO-H_2_O_2_-nitrite (NO_2_^−^) system and is often seen at sites of inflammation [17].

While MPO-generated oxidants are microbicidal and thus beneficial, these oxidants can also damage normal cells and tissues/proteins by excess production of respective halides [12,18]. There has been considerable evidence linking the production of HOCl with damage to host proteins, including DNA, RNA, lipids, and (lipo)proteins (reviewed in [19]). MPO is generally considered a myeloid specific gene with expression restricted to bone marrow myeloid precursors, but the human MPO (hMPO) gene can escape this restriction in some stress situations, such as in macrophages in cardiovascular disease [20,21,22,23,24,25], microglia, astrocytes and neurons in Alzheimer’s Disease (AD) [26,27,28] and in astrocytes and neurons in PD [29,30,31,32].

In order to study the effects of hMPO expression in mouse models of neurodegenerative diseases, we created a humanized mouse model transgenic for a single copy of the hMPO gene in a 32 kb restriction fragment [33]. When this mouse strain was crossed to the A53T mouse model of PD, the MPO gene was expressed in neurons [31]. This atypical expression of hMPO is thought to be due in part to the insertion in the promoter of an Alu element encoding several overlapping binding sites for members of the nuclear receptor superfamily of transcription factors (including retinoic acid receptor, thyroid hormone receptor, and peroxisome proliferator-activated receptor γ as well as SP1 [34,35,36,37]. A polymorphism in these Alu nuclear receptor sites, −463G, increases hMPO expression and has been linked to increased risk for AD [26,38,39,40,41,42], cardiovascular disease [20,21,22,23,24,25], lung cancer [43,44], and some epithelial cancers [45]. The murine MPO (mMPO) gene, lacking the primate-specific Alu moiety, is expressed at relatively low levels in mouse models of AD [27] or in the A53T-αSyn model of PD [31].

Within the halogenation process the MPO-H_2_O_2_ system uses SCN^−^ to form HOSCN [16]. HOSCN plays a beneficial role as a bacteriostatic agent and is a less powerful oxidizing agent and is more thiol specific than HOCl or HOBr [46,47,48]. The beneficial effects of SCN^−^ may stem from competition with Cl^−^ as a potential MPO substrate, thereby reducing HOCl formation in the presence of H_2_O_2_. Several animal studies suggest that SCN^−^ treatment may be useful therapeutically. A recent study used our humanized MPO mice crossed to low-density lipoprotein receptor knockout mice (Ldlr^--/-^) as a model for atherosclerosis [49]. Supplementation of SCN^−^ in the drinking water led to reduced oxidative damage by the MPO product HOCl. In mice treated with SCN^−^ there was a 2-fold increase in plasma SCN^−^ and a 26% reduction in atherosclerotic plaque area compared to control mice [49].

This result led us to investigate the effects of SCN^−^ in the hMPO-A53T mouse model of PD. Our findings demonstrate that treatment of mice with SCN^−^ improved the motor abilities on the rotarod, balance beam, and the wire hang in the hMPO-A53T mice as compared to nontreated hMPO-A53T mice. There was also less chlorination and nitration in the brains of the treated hMPO-A53T mice compared to the nontreated controls.

Importantly, the glymphatics system, a term used to describe the brain lymphatic vessels associated with glial cells (astrocytes), is a newly described network in the brain that is largely responsible for the removal of solutes and metabolic waste from the extracellular spaces in the brain (reviewed in Refs. [50,51]). In PD, a dysfunctional glymphatic system impaired by the oxidation and aggregation of proteins such as αSyn is likely to contribute to disease progression. Our previous studies showed that MPO contributes to the oxidation and aggregation of αSyn [31]. With the increasing interest in the role of glymphatics in neurodegenerative diseases, we set out to evaluate the role of MPO in the proper functioning of the glymphatic system through immunohistochemistry using a series of antibodies for glymphatic markers as well as antibodies to MPO oxidation products in the hMPO-A53T mouse model.

In a surprising discovery we noted that there was increased chlorination and nitration in enlarged glymphatic vessels colocalizing with glymphatic markers in the hMPO-A53T model. Interestingly, when these mice were treated with SCN^−^ there was a significant reduction in chlorination and nitration around the glymphatic vessels located near the ventricles, as well as a reduction in vessel size. This was accompanied by a significant improvement in the motor behavior tests. The results suggest that the inhibition of MPO may provide a therapeutic option for PD and other neurodegenerative diseases in which MPO has been implicated.

## 2. Materials and Methods

### 2.1. Reagents

DAPI (4′, 6-diamidino-2-phenylindole) (D-1306), the surfactants Tween-20 (P7949), NP40 (NP40S) and Triton X-100 (T8787), potassium cyanate, deoxyribonuclease I (D5025) were from Sigma. Vectashield™ mounting media (H-1000) was from Vector Labs. Non-immune donkey (017-000-121 or horse serum (008-000-121) were obtained from Jackson Immunoresearch Labs. Bovine serum albumin (BP1600100) as well as all general chemicals used for these studies were from Thermo Fisher, San Diego, CA, USA.

### 2.2. Antibodies

Primary antibodies used in this study included polyclonal antibodies (pAbs) such as rabbit anti-hMPO (DAKO A0398, 1/500 for immunohistochemistry (IHC) and goat anti-hMPO that is affinity purified against hMPO (R&D Systems, AF3174, 1/500 IHC). These antibodies have been used in a number of studies involving MPO including a recent report in which we showed the DAKO pAb rabbit and R&D goat MPO antibodies recognize MPO in immunoblots or immunostains of T47D cells transfected with a hMPO expression construct but did not recognize nontranfected T47D cells [52]. We generated our own rabbit pAb using purified neutrophil-derived MPO from Lee Biosciences as the antigen. Our rabbit pAb was used interchangeably with R&D goat pAb for immunofluorescence with no discernible difference in the results [52]. We further generated rabbit pAb against carbamylated αSyn (1:200 IHC). The characterization of the antibody has been previously described [31].

Other antibodies used in this study include monoclonal antibody (mAb) αSyn (Cell Signaling (Danvers, Ma, USA), D37A6, 1/500 IHC), mAb αSyn (BD Biosciences, San Diego, CA, USA, 6107877; 1/500 IHC), mAb anti-nitrated αSyn (Syn505, Life Technologies, San Diego, CA USA, 358300, 1/500 IHC), [8], mAb anti-αSyn raised against Lewy bodies which recognizes an epitope encoded by amino acids 115-122, and recognizing human but not mouse αSyn (LB509, Santa Cruz, Dallas, TX, USA, sc-58480, 1/100 IHC) [53], rabbit pAb anti-nitrotyrosine (nitroTyr, Millipore, St. Louis, MO, USA AB5411, 1/200 IHC), mAb anti-nitro-synuclein (recognizing Tyr^39^ clone nSyn14, Millipore-Sigma, 36-012 1/200 IHC) [54], mAbs raised against HOCl-modified proteins (clone 2D10G9) (1/50 IHC) have been previously validated [55,56], rabbit pAb against aquaporin 4 (Anti-AQP4, Sigma-Aldrich, St. Louis, MO, USA, HPA014784, 1/200 IHC), rabbit pAb against lymphatic vessel endothelial hyaluronan receptor (anti-LYVE 1 AB2988, EMD Millipore, Temecula, CA, USA 1/200 IHC). Secondary antibodies labeled with Alexa Fluor 488 (donkey anti-rabbit, A21206 and donkey anti-mouse, A21202) and Alexa Fluor 594 (donkey anti-rabbit, 21207 and donkey anti-mouse, A21203) were from Molecular Probes, goat or donkey anti-mouse HRP and anti-rabbit HRP-conjugated IgG were from Jackson ImmunoResearch, West Grove, PN, USA.

### 2.3. Immunohistochemistry (IHC)

Mice were sacrificed by exposure to CO_2_, then transcardially perfused with ice cold phosphate-buffered saline (PBS). Mouse brain tissue was fixed overnight in 4% paraformaldehyde in PBS. Free floating (40 microns) sagittal or coronal sections, obtained with a Leica VT1000S vibrating microtome, were incubated with 10% H_2_O_2_ in PBS for 10 min, blocked with 10% non-immune goat serum for 12 h, and incubated overnight at 4 °C with primary antibodies in the presence of non-immune serum from the secondary antibody host [57,58]. Primary antibodies were detected with biotinylated secondary antibodies and avidin-conjugated horseradish peroxidase (Vectastain ABC kit, Vector Laboratories, Burlingame, CA, USA) and detected with peroxidase chromogen (SG (peroxidase substrate) or 3-amino-9-ethylcarbazole, Vector Laboratories). Nonfluorescent immunostaining of paraffin sections was carried out with primary antibodies in PBS + 0.05% Tween 20 (PBST) with 10% non-immune donkey serum, followed by biotinylated secondary donkey antibodies (Vector) (1:200, 1 h) and avidin–biotin conjugates (Vector Elite ABC system) (1:200, 2 h), and developed with peroxidase substrate. Images were obtained with an Olympus BX51 microscope with 20×, 40×, and 100× objectives. All sections were processed simultaneously under the same conditions and each experiment was repeated at least three times with multiple biological replicates.

Paraffin-embedded sections of mouse brains were cleared by xylene and ethanol prior to heat induced antigen retrieval in 10 mM sodium citrate buffer, 0.05% Tween 20, pH 6.0. Sections were incubated in 10% normal donkey serum for 1 h, followed by incubation for 12 h in primary antibodies in PBST and 10% non-immune donkey serum. Following incubation, the sections were washed in PBST for 2 h prior to incubation with secondary fluorescent donkey antibodies conjugated to Alexa Fluor 488 (green) or Alexa Fluor 594 (red) at 1:3000 dilution for 1 h. Images were obtained on a conventional fluorescent microscope (Olympus BX51). Images were saved as tiff files in 8-bit per channel format (24-bit RGB) and processed with Photoshop for assembly of figures. All images presented here are representative of at least three independent immunostaining experiments.

### 2.4. Quantitative Immunohistochemical Analysis

Immunostained sections were analyzed with a digital Olympus bright-field and fluorescence microscope (BX51). For each analysis, a minimum of five mice of each genotype were used. For each mouse, a minimum of three paraffin sections were analyzed. In each section, four areas of interest were imaged. Quantitation of immunoreactivity was determined by optical density analysis using ImageJ/FIJI software. Levels of optical density were corrected to background using sections that lacked exposure to the primary antibody. After correction to background, the levels of immunoreactivity were expressed as corrected optical density relative units. Statistical analysis was conducted using GraphPad Prism software (v9) and Student’s *t*-test for comparing the means of two samples or one-way ANOVA with posthoc Dunnet when comparing the hMPO-A53T, hMPO, and A53T transgenic mice versus wildtype (WT, C57Bl/6) animals.

### 2.5. Animals

All animal work was carried out at the AAALAC accredited animal facility at the Sanford-Burnham-Prebys Medical Discovery Institute. The care of the mice, and all the procedures performed were approved by the Institutional Animal Care and Use Committee (protocol number 15-097) and complied with National Institutes of Health animal care guidelines.

### 2.6. Transgenic Mice

Transgenic mice carrying the human −463G MPO allele have been previously described [32,33,35,59]. There is one copy of the hMPO −463G allele in the mouse genome on the X chromosome. The mice were generated by microinjection of a 32kb BST11071 restriction fragment into C57BL6/J eggs [33]. Male mice were used for all these studies due to the fact that the hMPO transgene is on the X chromosome resulting in random inactivation of one MPO allele on an X chromosome in female mice. These mice are currently available from Jackson Labs (Tg(MPO*-463G)1Wfr/J) (Jackson 035208). All experiments were performed with mice hemizygous for the hMPO or αSyn transgenes. Mice overexpressing the human A53T αSyn mutant gene under control of the human thymus cell antigen 1 (Thy1) promoter (THY1-SNCA*A53T)M53SUD/J) (Jackson, 008135) have been described [60,61,62]. The primers for geno-typing the mice are: hMPO for: 5′-GCAATGGTTCAAGCGATTCTT-3′; hMPO rev: 5′-CGGTATAGGCACACAATGGTGAG-3′; hSNCA for: 5′-GGCACCTAGAGGATCTCGACTAGTGG-3′; Thy1-SNCA rev: 5′-GATG ATGGCATGCAGCACTGG-3′.

### 2.7. Behavior Tests

#### 2.7.1. Wire Hanging Test

The mouse was placed on the top of a standard cage lid that was then shaken gently to induce the mouse to grip the wires, and then inverted for up to 60 s. The lid was elevated 3 ft over soft bedding. Three consecutive trials were performed with a 5 min resting period between each trial. The latency to fall from the cage lid was recorded. Behavior data were analyzed using a one-way analysis of variance (ANOVA) followed by Dunnets post hoc test using GraphPad Prism v9. SCN^−^ treatment began at weaning at 21 days. Trials were performed at ages between 40 to 50 days.

#### 2.7.2. RotaRod Test

The test assesses motor coordination of mice placed on a five-lane rotating rod (San Diego Instruments, CA, USA) that accelerates slowly from 2 to 20 rpm over a period of 300 s. The instrument records the latency to fall and the rotator speed at fall. The mice were trained on the device for 5 min at a constant speed of 5 rpm for 3 days prior to the experiment. In the experiment, the mice were placed on the RotaRod for up to 300 sec for three consecutive trials with a 5 min rest between trials. Behavior data were analyzed using a one-way analysis of variance (ANOVA) followed by Dunnets post hoc test using GraphPad Prism v9. SCN^−^ treatment began at weaning at 21 days. Trials were performed at ages between 50 to 60 days.

#### 2.7.3. Balance Beam

Mice were trained to walk across an elevated round beam of 1 m length and 1 cm diameter. The beam was elevated 3 ft above the bench with soft padding placed below. Animals were trained three times on the beam one day before the test. All mice were given three consecutive trials. If a mouse paused on the beam it was gently touched on the hindquarters to encourage movement. If the animal fell off the beam the timer was paused and the animal was placed back on the beam at the position it had when it fell. Between each mouse trial, the beam was cleaned with water. Behavior data were analyzed using a one-way analysis of variance (ANOVA) followed by Dunnets post hoc test using GraphPad Prism v9. SCN^−^ treatment began at weaning at 21 days. Trials were performed at ages between 50 to 60 days.

#### 2.7.4. Statistical Analysis

All experiments were performed blinded. All values are expressed as the mean ± S.E.M. Statistical differences were considered significant at *p* < 0.05 level. All analyses were performed using GraphPad Prism v9. Behavior data were analyzed using a one-way analysis of variance (ANOVA) followed by a Dunnets post hoc test or the *t*-test.

## 3. Results

### 3.1. Motor Behavior

To determine if treating mice with SCN^−^ had effects on motor activity in the hMPO-A53T model we carried out several standard motor behavior tests. Mice heterozygous for the hMPO and A53T transgenes were used in the behavior assays to ensure that there were equivalent copy numbers of each transgene. Our earlier study [31] showed that insertion of a single copy of the hMPO transgene in the A53T model of PD led to exacerbation of PD-like brain oxidative damage and greater motor impairment. The hMPO-A53T mice exhibited worse motor abilities than the A53T mice on the balance beam, wire hang, and rotarod [31]. Moreover, the impaired motor abilities in hMPO-A53T mice correlated with greater oxidative damage in the brain.

MPO reacts with Cl^−^ in the presence of H_2_O_2_ to generate HOCl, a toxic chlorinating agent. SCN^−^ is a more favorable substrate of hMPO, and generates the less toxic carbamylated lysine, which can be reversible, unlike HOCl-modified epitopes. In this study, we investigated whether SCN^−^ treatment of hMPO-A53T mice would compete with Cl^−^, resulting in less Cl^−^ oxidation and less motor impairment. At 21 days of age, the hMPO-A53T and A53T mice were treated or not treated with SCN^−^ for 40 to 60 days. Motor abilities were assessed in three behavior tests, time required to traverse the balance beam, ability to support their weight holding onto a wire mesh, and ability to maintain balance on a rotating rod.

#### 3.1.1. Balance Beam

**Figure 1:**
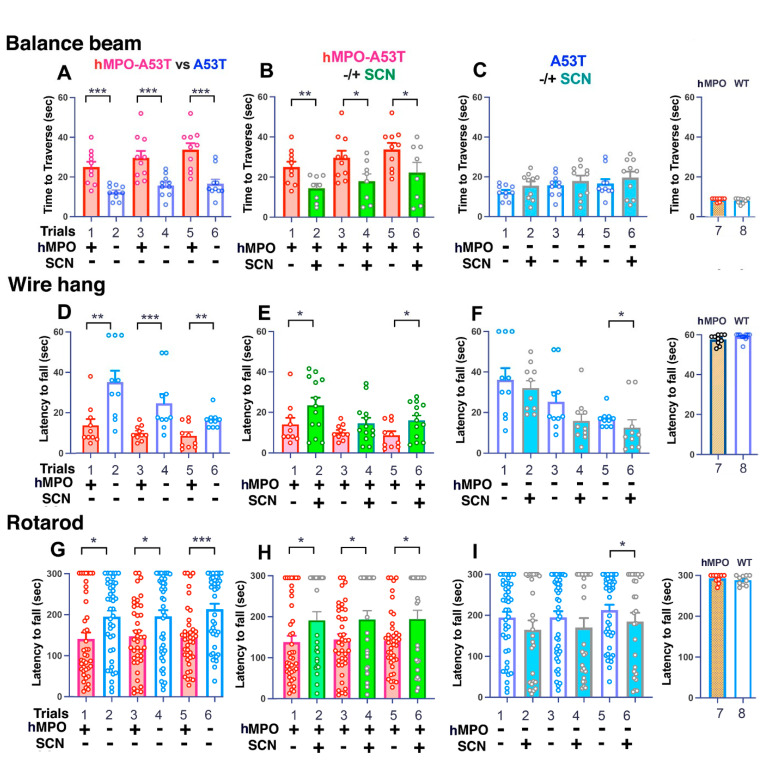
Impaired motor abilities in hMPO-A53T mice compared to A53T. (**A**) Balance beam analysis was performed with genotypes hMPO-A53T (n = 10) (lanes 1,3,5) versus A53T (n = 10) (lanes 2,4,6). Only male mice were used in these experiments. Three consecutive trials were performed for each group with rest intervals of 5 min. (**B**) hMPO-A53T (n = 10) (lanes 1,3,5) vs. hMPO-A53T SCN^−^ treated (n = 8) (lanes 2,4,6). (**C**) A53T (n = 10) (lanes 1,3,5) vs. A53T SCN^−^ treated (n = 10) (lanes 2,4,6). hMPO mice (n = 10) (lane 7) vs. WT (C57Bl/6) (n = 9) (lane 8). Only the third trial is shown for WT and hMPO mice. Trials A lanes 1,3,5 are the same as B lanes 1,3,5. Trials A lanes 2,4,6 are the same as C lanes 1,3,5. (**D**–**F**) The wire hang was performed with the indicated genotypes as in panels A,B,C (n = 10 A53T, n = 10 hMPO-A53T, n = 10 hMPO, n = 10 WT). Three trials with rest intervals were performed for each group. Only the third trial is shown for WT and hMPO mice. (**G**–**I**) Rotarod analysis was performed with the genotypes indicated in panels A,B,C (n = 39–44 for each group, n = 10 hMPO, n = 10 WT). Three consecutive trials were performed for each group with rest intervals of 5 min. Only the third trial is shown for WT and hMPO mice. Behavior data were analyzed using a one-way analysis of variance (ANOVA) followed by Dunnets post- hoc test using GraphPad Prism v9. Data are represented as mean +/− S.E.M. (* *p* < 0.01, ** *p* < 0.005, *** *p* < 0.001).

The balance beam is a test of balance and motor impairment (Figure 1). Mice are required to traverse a dowel (1 m) with a diameter of 1 cm. Consistent with our prior study, hMPO-A53T required significantly more time to traverse the beam (Figure 1A, red bars) (scores 25, 30, 34 s) than A53T (Figure 1A, white bars) (scores 12, 16, 17 s). This indicates a functional interaction between hMPO and αSyn to increase motor impairment at these early stages of αSyn mediated damage. WT mice (Figure 1C, trial 8) or hMPO transgenic mice lacking the A53T gene (Figure 1C, trial 7) were able to traverse the beam much faster than A53T (scores 10 and 9 s, respectively).

When the hMPO-A53T mice were either treated or not treated with SCN^−^, the treated hMPO-A53T mice were able to cross the beam more rapidly (Figure 1B, green bars) (scores 14, 18, 22 s) than untreated hMPO-A53T mice (Figure 1B, red bars) (scores 25, 30, 34 s). Interestingly, the SCN^−^ treated hMPO-A53T scores (Figure 1B, green bars), scores 14, 18, 22 s) were similar to the scores of A53T lacking hMPO in panel A (Figure 1A, white bars) (scores 12, 16, 17 s), indicating that SCN^−^ counteracts the deleterious effects of hMPO in the hMPO-A53T model.

When the A53T mice were treated or untreated with SCN^−^ the treatment did not alter the traversal times for A53T mice (Figure 1C, blue bars), scores 16, 18, 20 s) versus untreated A53T (Figure 1C, white bars) (scores 12, 16, 17 s), indicating the beneficial effect of SCN^−^ in hMPO A53T mice is dependent on the presence of the hMPO transgene.

#### 3.1.2. Wire Hang

The wire hang is a method to test grip strength, stamina, and neuromuscular dysfunction. The mice are required to support their weight inverted on a cage top for up to 60 s for three consecutive tests with 5 min rest intervals. In most cases, WT or hMPO mice easily maintained grip through the three 60 s trials (mean latency to fall of 56 s) (Figure 1F, lanes 7 and 8). The A53T mice (Figure 1D, white bars) fell at a mean of 36 s on the first trial and earlier on succeeding trials. The hMPO-A53T (Figure 1D, red bars) fell earlier at a mean of 14 s, and still earlier on successive trials.

When the hMPO-A53T mice were either treated or not treated with SCN^−^, the treated hMPO-A53T mice were able to hold onto the wire cage longer (Figure 1E, green bars), (scores 23, 15, 16 s) than the untreated hMPO-A53T mice (Figure 1E, red bars) (scores 14, 10, 9 s). The scores of the SCN^−^ treated hMPO-A53T mice (Figure 1E, green bars) thus approach the scores of the untreated A53T mice (Figure 1D, white bars) (scores 36, 25, 17 s) indicating that SCN^−^ enhances motor abilities by counteracting the effects of the hMPO gene. The hMPO gene expresses higher levels of MPO in the brain than mouse MPO, resulting in high levels of HOCl, a potent oxidant. SCN- is a more favored substrate for MPO than Cl-, thus SCN- treatment reduces production of HOCl, thus counteracting the deleterious oxidative effects of hMPO in the hMPO-A53T model, resulting in improved motor abilities.

When the A53T mice were either treated or not treated with SCN^−^, both groups showed a gradual reduction in ability to hold on to the wire on the three consecutive trials (Figure 1F). The treated A53T mice were slightly less able to maintain hold on the wire cage (Figure 1F, blue bars) (scores 32, 16, 12 s) as compared to untreated A53T (Figure 1F, white bars) (scores 36, 25, 17 s). This indicates that SCN- treatment has a mild deleterious effect in the absence of hMPO expression. The A53T mice do express mouse MPO which reacts with SCN- to produce the oxidant HOSCN which oxidizes many proteins, including mitochondrial proteins, which could result in the reduced ability to maintain hold on the wire. MPO reacts with Cl- to produce the more toxic HOCl. Treatment of the hMPO-A53T mice with SCN- would generate HOSCN thereby competing against production of the more toxic HOCl-, thereby enhancing motor abilities.

#### 3.1.3. Rotarod

The accelerating Rotarod tests balance, coordination, muscle strength, and stamina as the mice are required to maintain balance on a rotating bar that accelerates from 2 to 20 rpm over a period of 300 s. Mice at ages between 50 and 60 days were tested on the rotarod with three consecutive tests of up to 300 s with a 5 min rest interval (Figure 1G–I). The WT and hMPO control mice were able to maintain balance for most of the 300 s test period (Figure 1I, trials 7 and 8).

The A53T mice (Figure 1G, white bars) were less able than controls to maintain balance resulting in a mean latency to fall of 194 s on trial 1, while the hMPO-A53T mice (Figure 1, Panel G, red bars) were less able to maintain hold with a mean latency of 130 s on trial 1. These findings show that the hMPO transgene exacerbates the motor impairment of the A53T mice at these early ages. Importantly, the hMPO transgene had no effect on motor abilities in the absence of the A53T gene (compare WT; Figure 1I, trial 8 and hMPO; Figure 1I, trial 7), indicating that hMPO synergizes with αSyn to exacerbate motor impairment.

When the hMPO-A53T mice were either treated or not treated with SCN^−^, the treated hMPO-A53T mice were able to stay on the rotarod longer (Figure 1H, green bars) (scores 194, 196, 197 s) than the untreated hMPO-A53T mice (Figure 1H, red bars) 140, 147, 151 s). The scores of the treated hMPO-A53T mice (Figure 1H, green bars) (scores 194, 196, 197 s) were similar to the untreated A53T mice (Figure 1G, white bars) (scores 194, 195, 213 s) indicating that SCN^−^ counteracts the deleterious effects of hMPO in the hMPO-A53T model.

When the A53T mice were treated or untreated with SCN-, SCN- treatment again moderately impaired the ability to stay on the rotarod (Figure 1I, blue bars) (scores 165, 170, 185 s) as compared to untreated A53T (Figure 1I, white bars) (scores 194, 195, 212). Thus, SCN- slightly impairs motor abilities in A53T mice lacking hMPO. As in panel F, this is likely due to expression of mouse MPO which reacts with SCN- to produce HOSCN which oxidizes many proteins, likely contributing to motor impairment.

In conclusion, SCN^−^ treatment enhanced motor abilities of the hMPO-A53T mice, but not the A53T mice lacking hMPO. SCN- had no effect on the hMPO mice lacking A53T or the A53T control mice lacking hMPO.

### 3.2. MPO, MPO-Generated HOCl Epitopes and LYVE1 Colocalize in Vessels around the Lateral Ventricles in the hMPO-A53T Mouse

Immunostaining was performed to investigate the mechanism by which SCN^−^ reverses the deleterious impact of hMPO in the A53T model. To identify sites modified by the hMPO-H_2_O_2_-Cl^−^ system we used antibodies to MPO along with mAb 2D10G9 that specifically recognizes HOCl modified epitopes/proteins [56].

Sagittal sections from hMPO-A53T brain were probed with 2D10G9 and antibodies to LYVE1, a marker for LYmphatic VEssels, also termed glymphatic vessels (glia-lymphatics) in the brain referring to the astrocytic end feet with AQP4 water channels that encase the blood vessels creating peri-arterial channels that convey cerebral spinal fluid (CSF) into the brain parenchyma and peri-venous channels that carry waste such as oxidized αSyn out of the parenchyma.

**Figure 2:**
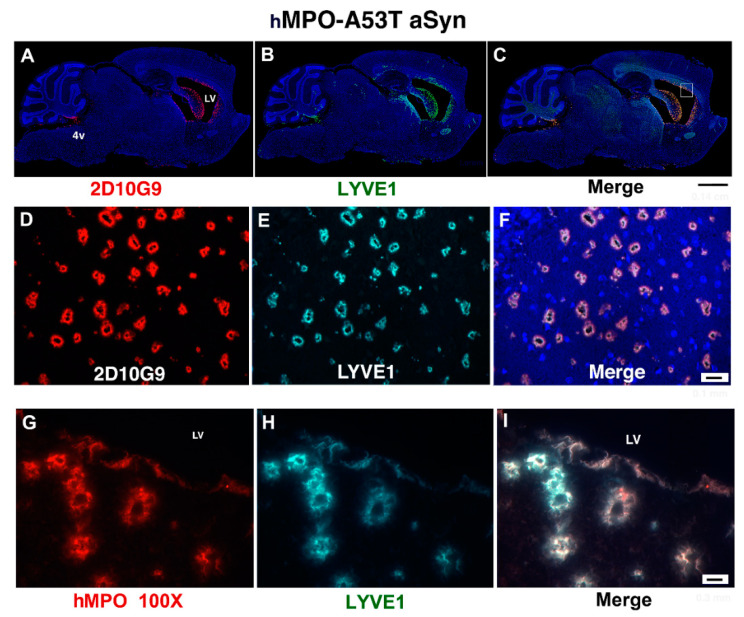
Antibodies to hMPO, 2D10G9 (detects HOCl modified epitopes), and LYVE1 (glymphatic vessel marker) colocalize in dystrophic vessels bordering the lateral ventricles in the hMPO-A53T mouse brain. (**A**–**C**) Immunofluorescence staining of a whole mount sagittal section of hMPO-A53T mouse brain shows colocalization of 2D10G9 ((**A**), Alexafluor 594, red) and LYVE1 ((**B**), Alexafluor 488, green) and the merged image in Panel (**C**). Immunofluorescence is visible in vessels around the lateral ventricles (LV), and 4th ventricle under the cerebellum. The box (**C**) shows an example of location of vessels as seen in (**D**–**F**). Scale bar: (**A**–**C**) 1.5 mm. (**D**–**F**) Higher magnification (20×) of these regions shows the vessels around the ventricle stained for 2D10G9 ((**D**), Alexafluor 594, red) and LYVE1 glymphatic/lymphatic vessel marker ((**E**), Alexafluor 488, green) and the merged image (**F**). Scale bar: (**D**–**F**) 0.1 mm. (**G**–**I**) Higher magnification of panels of (**D**–**F**) (100×, oil immersion) shows the atypical structure of the vessels which have lumens surrounded by walls of filamentous and granular components. Some vessels appear to open to the lateral ventricle (LV). Scale bar: (**G**–**I**) 0.03 mm.

In sagittal sections (Figure 2A–C), 2D10G9 and LYVE1 antibodies colocalized in groups of blood/lymph vessels located around the perimeter of the lateral ventricles (LV) and 4th ventricle (4v). Higher magnification (20×, Figure 2D–F) revealed that these vessels have a thick vessel wall colocalizing with 2D10G9 and LYVE1 antibodies. Lumens are clearly visible in these vessels. The same staining pattern was observed with antibodies to MPO and LYVE1. At greater magnification (100×), MPO (Figure 2G) and LYVE1 (Figure 2H) were found to colocalize in the vessel walls which appear irregular rather than smooth, comprised of filamentous material with some granularity (Figure 2G–I). Some of these vessels (upper right in I) appear to merge with the ependymal cell lining of the ventricle. The periventricular location of these atypical LYVE1 positive vessels suggests a connection to the glymphatic system of waste removal from the brain.

### 3.3. SCN^−^ Treatment Reduces the Size and Number of 2D10G9 MPO Modified HOCl Epitopes in the hMPO-A53T Mice but Not in A53T Lacking the hMPO Transgene

**Figure 3:**
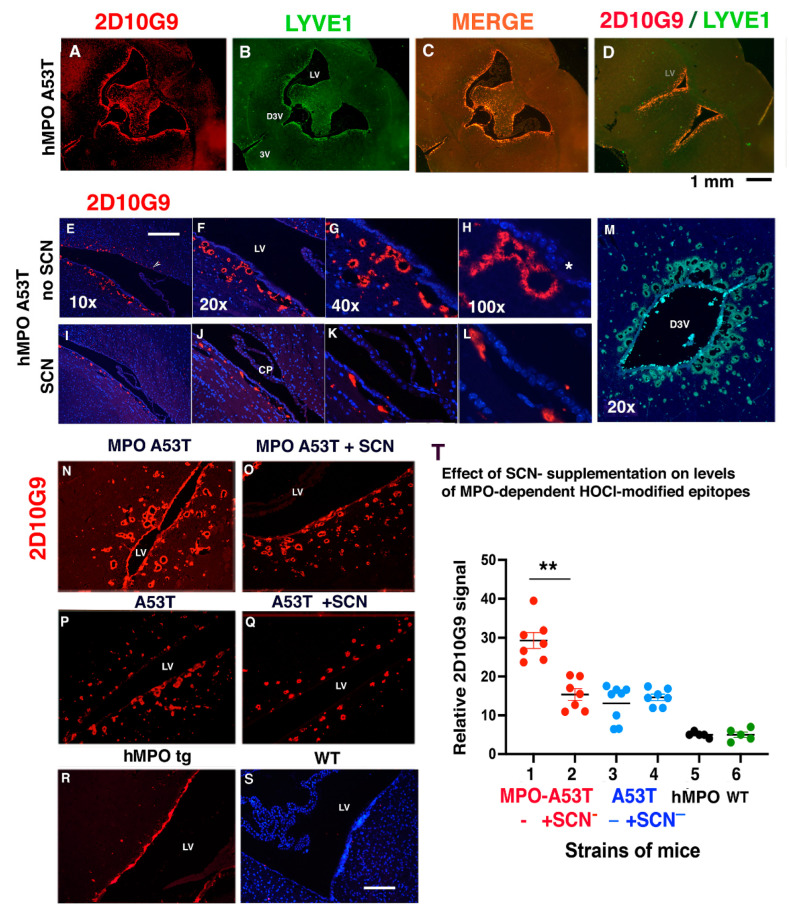
Thiocyanate treatment reduces size and number of 2D10G9 MPO modified HOCl epitopes in the MPO A53T mice but not in A53T lacking hMPO. (**A**–**D**) Coronal sections of hMPO-A53T brain show colocalization of MPO-generated HOCl epitopes (using 2D10G9) (**A**) and LYVE1 (**B**) with merged image in (**C**) and another merged image from a more anterior position (**D**). Panel (**B**) shows location of the lateral ventricles (LV), dorsal third ventricle (D3V) and third ventricle (3V). (**M**) 2D10G9 staining of numerous vessels surrounding the dorsal third ventricle. Scale bar (**A**–**D**) 1 mm. (E-H) 2D10G9 immunostaining of periventricular vessels in hMPO-A53T brain from mice that were not treated with SCN^−^. Images were made at 10× (**E**), 20× (**F**), 40×, (**G**), and 100× (**H**) objectives. Scale bar for E–L: E 0.1 mm. (**I**–**L**) Immunoreactivity of 2D10G9 in periventricular vessels in hMPO-A53T brain from mice that were treated with SCN^−^. Images were made at 10× (**E**), 20×, (**F**) 40× (**G**), and 100× (H) objectives. Choroid Plexus (CP, Panel (**J**)). (**N**–**Q**) 2D10G9 immunofluorescence staining of hMPO-A53T lateral ventricle from mice not treated with SCN^−^ (**N**), compared to hMPO-A53T mice treated with SCN^−^ (**O**). D10 staining of A53T mice not treated with SCN^−^ (**P**), compared to A53T mice treated with SCN^−^ (**Q**). Controls show D10 staining of the lateral ventricle from MPO transgenic mice lacking the A53T transgene (**R**) and WT mouse brain (**S**). Objective was 20×. Scale bar: (**N**–**S**) 0.1 mm. (**T**) 2D10G9 immunofluorescence staining was quantitated for the periventricular regions from hMPO-A53T and A53T brain with or without SCN^−^ treatment. Lane 1 shows the relative 2D10G9 fluorescence signal in hMPO-A53T mice not treated with SCN^−^ (n = 7). Lane 2 shows the signal for hMPO-A53T mice treated with SCN^−^ (n = 7). Lane 3 shows the signal in A53T mice not treated with SCN^−^ (n = 8). Lane 4 shows the signal in A53T mice treated with SCN^−^ (n = 7). Little to no signals were obtained for sections of hMPO transgenic mouse brain lacking A53T (lane 5) or WT mouse brain (lane 6). Statistical analysis was conducted in Panel T using GraphPad Prism software (v9) and Student’s *t*-test for comparing the means of two samples (*p* < 0.01 **).

Coronal sections of hMPO-A53T brain revealed colocalization of HOCl-modified epitopes (Figure 3A) and LYVE1 (Figure 3B, merged in 3C and another example in 3D) in vessels surrounding the LVs, as well as the dorsal third ventricle (D3V) and third ventricle (3V) (Figure 3B,M). To gain insight into the mechanisms by which SCN^−^ reduces the impact of MPO on motor dysfunction, we analyzed the peri-ventricle vessel morphology in hMPO-A53T mice that were not treated with SCN^−^ (Figure 3E–H) versus hMPO-A53T mice treated with SCN^−^ (Figure 3I–L) at several magnifications (10× through 100×). In hMPO-A53T mice lacking SCN^−^ treatment (Figure 3E–H), there were greater numbers of HOCl-modified vessels (2D10G9) around the ventricles, and the vessels had larger lumens. Some of the vessels appear to be in contact with the ependymal layer (DAPI, blue) and some may open to the ependymal layer (Figure 3H, asterisk). In hMPO-A53T mice that had been treated with SCN^−^ (Figure 3I–L) there were fewer 2D10G9 positive vessels (red) and the vessels were smaller and generally lacked lumens suggesting the vessels had collapsed.

Quantitation was carried out to measure the HOCl-modified epitopes in the peri-ventricular regions from hMPO-A53T mice without SCN^−^ treatment (Figure 3N, n = 7) versus hMPO-A53T with SCN^−^ treatment (Figure 3O, n = 8) (Figure 3, Panel T, lanes 1 and 2). SCN^−^ significantly reduced the 2D10G9 immunoreactivity in peri-ventricular vessels in the hMPO-A53T mice (Figure 3T, lane 2), as compared to hMPO-A53T lacking SCN^−^ treatment (Figure 3T, lane 1). In contrast, there was no significant effect of SCN^−^ on 2D10G9 signal in A53T mice with SCN^−^ (Figure 3Q) (Figure 3T, lane 4) or without SCN^−^ treatment (Figure 3P) (Figure 3T, lane 3). The peri-ventricular vessels were not detected by 2D10G9 in the hMPO mouse brain lacking the A53T transgene (Figure 3R) (Figure 3T, lane 5) nor in WT mouse brain (Figure 3S) (Figure 3T, lane 6), indicating that A53T transgene is required for these atypical peri-ventricular vessels.

### 3.4. SCN^−^ Treatment of hMPO-A53T Mice Reduces Levels of Nitrated αSyn in Glymphatic Vessels Surrounding the Ventricles

**Figure 4:**
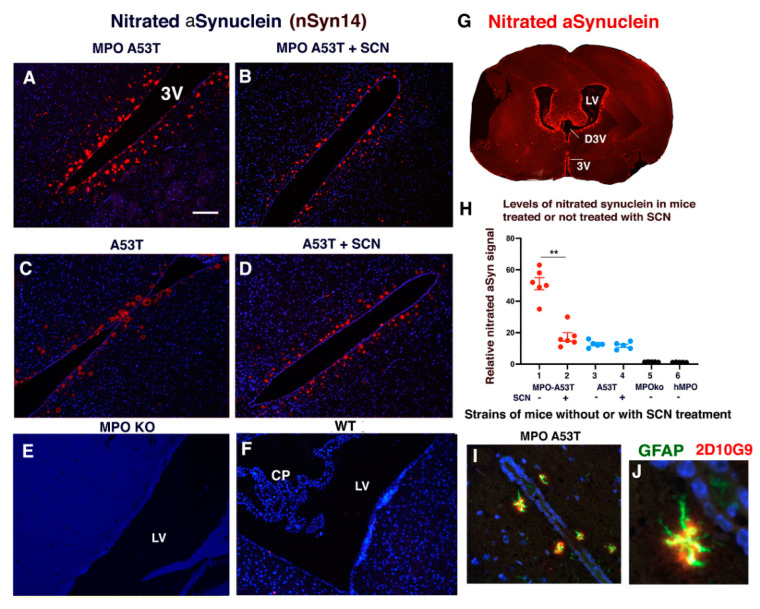
Thiocyanate treatment of hMPO-A53T mice reduces levels of nitrated aSyn in glymphatic vessels surrounding the ventricles. Immunostaining with antibodies to nitrated αSyn (nSyn14) was performed on brain sections from hMPO-A53T and A53T mice that had been treated (**B**,**D**) or not treated (**A**,**C**) with SCN^−^. Higher levels of nSyn14 staining were detected in vessels in hMPO-A53T brain lacking SCN^−^ treatment (**A**) than hMPO-A53T mice treated with SCN^−^ (**B**). A53T brain lacking hMPO (**C**) had less nSyn14 reactivity than hMPO-A53T brain (**A**). There was no difference in levels of nSyn14 reactivity in A53T brain with SCN^−^ treatment (**D**) or without SCN^−^ treatment (**C**). As controls, there were no significant 2D10G9 staining in brains of hMPO KO (C57Bl/6) (**E**) or WT mice (**F**). Scale bar 0.1 mm. (**G**). Coronal section of hMPOA53T brain immunostained for nitrated aSyn in vessels surrounding the lateral ventricles (LV), dorsal third ventricle (D3V) and third ventricle (3V). (**H**) Levels of nitrated αSyn detected by nSyn14 antibody and Alexafluor594, red in areas around the third ventricle. Numbers of mouse brains examined were 5 to 7 for each genotype and treatment. Lane 1 shows hMPO A53T mice not treated with SCN^−^. Lane 2 shows hMPO-A53T mice that had SCN^−^ treatment. Lane 3 shows A53T mice that lacked SCN^−^ treatment. Lane 4 shows A53T mice that had SCN^−^ treatment. Control lane 5 shows MPO KO (C57Bl/6) mice that lacked SCN^−^ treatment. Lane 6 shows hMPO mice that lacked SCN^−^ treatment. Statistical analysis was conducted in Panel H using GraphPad Prism software (v9) and Student’s *t*-test for comparing the means of two samples (*p* < 0.01 **). (**I**) hMPO-A53T brain was immunostained for GFAP astrocyte marker (green) and 2D10G9 HOCl oxidized epitopes(red) alongside the 3rd ventricle. One vessel is enlarged in (**J**) showing astrocytic processes indicative of astrocytes.

Nitrated αSyn is an established marker of PD pathology. Our prior studies of the hMPO-A53T model showed increased nitration of αSyn in dystrophic neurons using mAb nSyn14 [31]. Here, we investigated whether hMPO expression in A53T brain led to increased levels of nitrated αSyn in the periventricular glymphatic vessels. We focused on the vessels around the 3rd ventricle (Figure 4G and Figure 3V). Immunostaining was carried out on brain sections from hMPO-A53T mice not treated with SCN^−^ (Figure 4A) or treated with SCN^−^ (Figure 4B), as well as A53T mice that were not treated with SCN^−^ (Figure 4C) or treated with SCN^−^ (Figure 4D).

Fluorescence quantification (Figure 4H) revealed higher levels of nSyn14 staining in vessels around the 3rd ventricle (Figure 4G and Figure 3V) in hMPO-A53T brain lacking SCN^−^ treatment (Figure 4A,H lane 1) than hMPO-A53T treated with SCN^−^ (Figure 4B,H lane 2). A53T brain lacking hMPO (Figure 4C,H lane 3) had less nSyn14 reactivity than hMPO-A53T brain (Figure 4A, and 4H lane 1), and A53T brain showed no difference in levels of nSyn14 reactivity with (Figure 4C,H lane 4) or without SCN^−^ treatment (Figure 4D,H lane 3). In controls, there was no significant nSyn14 staining in brains of mouse MPO^−/−^ (C57Bl/6 background) (Figure 4E,H lane 5) or WT (C57Bl/6) mice (Figure 4F,H lane 6).

Glymphatic vessels are encased in astrocytic end feet with AQP4 water channels enabling transport of CSF/water from the ventricles through astrocytes and into the parenchyma. To determine if astrocytes are present in these vessels, we stained the 3rd ventricle region with GFAP astrocytic marker along with 2D10G9 HOCl modified epitopes (Figure 4I,J). We detected astrocytes with typical elongate processes staining for GFAP (green) along with HOCl modified epitopes (2D10G9, red).

### 3.5. Glymphatic Vessels Colocalizing for MPO, LYVE1, AQP4, GFAP, 2D10G9, ntSyn14, and Carbamylated αSyn Can Be Toxic to Ependymal Cells Lining the Third Ventricle in hMPO-A53T Mice

**Figure 5:**
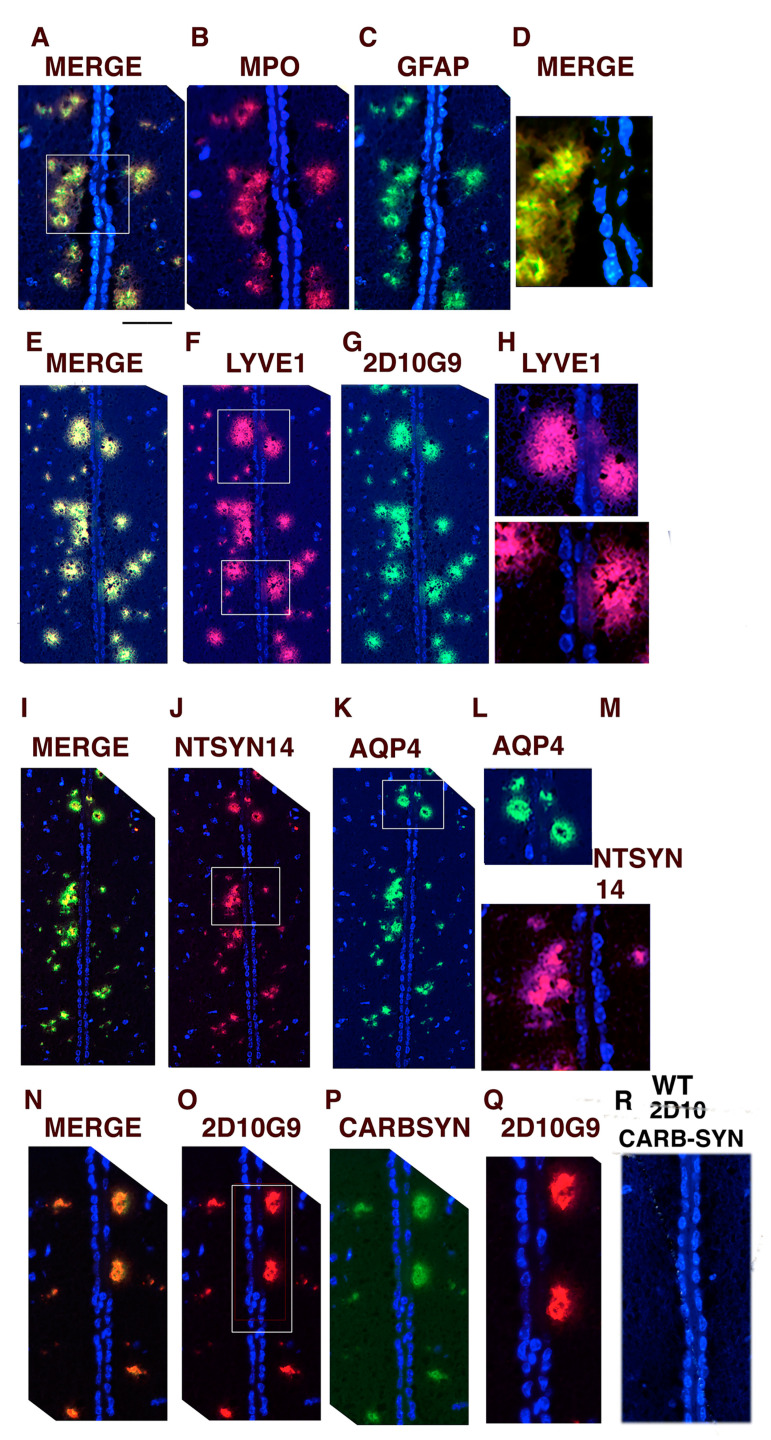
Colocalization for LYVE1, AQP4, MPO, GFAP, ntSyn14, HOCl oxidized epitopes, and carbsyn in periventricular vessels can be toxic to ependymal cells in hMPO-A53T mice. (**A**–**D**) Panels (**A**–**D**) show colocalization of MPO (**B**) and GFAP (**C**), marker for activated astrocytes, with merged image in (**A**) in glymphatic vessels alongside the ependymal cell nuclei of the 3rd ventricle. Scale bar 0.01 mm. The boxed area in (**A**) is enlarged in (**D**) to show loss of ependymal DAPI stained nuclei adjacent to the glymphatic vessels. (**E**–**H**) Panels E through H show colocalization in the glymphatic vessels of LYVE1 (**F**), marker of glymphatic vessels and 2D10G9 HOCl modified epitopes (**G**) with merge in (**E**). The boxed areas in (**F**) are enlarged in (**H**) to show loss of ependymal DAPI stained nuclei next to vessels. The scale is the same as in Figure 5A. Panels (**I**) through (**M**) show colocalization of nitrated aSyn (NTSYN14) and AQP4, marker of astrocytic end feet that encase glymphatic vessels, with merged image in (**I**). The boxed area in (**J**) and in (**K**) are enlarged in (**M**) and (**L**), respectively, to show loss of ependymal nuclei close to the vessels. The scale is the same as in Figure 5A. Panels (**N**–**Q**) show colocalization of 2D10G9 HOCl modified epitopes and carbamylated αSyn (carbsyn), a marker of hMPO oxidation. The boxed area in (**O**) is enlarged in (**Q**) to show loss of DAPI stained ependymal nuclei near the vessels. (**R**) shows lack of staining for 2D10G9 and carb-syn around control wildtype (WT) brain which exhibits healthy DAPI stained nuclei in the ependymal nuclei. Objective 20×. Scale bar under A, 0.01 mm.

The 3rd ventricle is situated at the base of the mouse brain (Figure 4G) and can appear in coronal sections as two closely associated parallel rows of ependymal cell nuclei. In the hMPO-A53T model, the 3rd ventricle (3V) is associated with the unusual glymphatic vessel structures on both sides (Figure 5). Figure 5A shows colocalization of MPO (B) and GFAP (C), a marker of activated astrocytes, in vessels that appear to be loosely organized, most lacking a clear lumen while some have a number of small lumens. Interestingly, when these abnormal vessels are located close to the ependymal cells, there is loss of the DAPI stained nuclei at that location as shown in the merged image of MPO/GFAP staining in Figure 5D.

Analogous findings were observed for the same 3V region costained for LYVE1 (Figure 5F) and 2D10G9 HOCl-epitopes (Figure 5G), merged in Figure 5E. Boxed areas of LYVE1 staining (Figure 5F), shown at greater magnification in Figure 5H, similarly show loss of DAPI staining of ependymal nuclei in close proximity to the vessels. The small holes or fenestrations in these vessel regions are more visible in these LYVE1 or 2D10G9 stained sections (Figure 5H). Costaining for ntSyn14 (Figure 5J) and AQP4 (Figure 5K, merged in I)) similarly showed loss of ependymal nuclei proximal to the vessels (enlarged in Figure 5L,M). Complete loss of DAPI stained ependymal nuclei was also observed for 2D10G9 staining (Figure 5O, enlarged in Figure 5Q) as well as carbamylated αSyn (CARBSYN) (Figure 5P). Figure 5R shows DAPI stained 3rd V nuclei from WT mouse brain that show absence of 2D10G9 and carbsyn staining, providing evidence that MPO and αSyn A53T must be present to elicit these atypical glymphatic vessels.

The confluence of these several markers associated with the glymphatic system (GFAP, AQP4, LYVE1) and PD (MPO, HOCl-modified epitopes, carbsyn, ntSyn14) argue that MPO oxidants may be an important contributor to the impairment of the glymphatic system in PD.

### 3.6. AQP4 and HOCl-Epitopes in Periventricular Vessels Suggest MPO Contributes to Glymphatic Impairment

**Figure 6:**
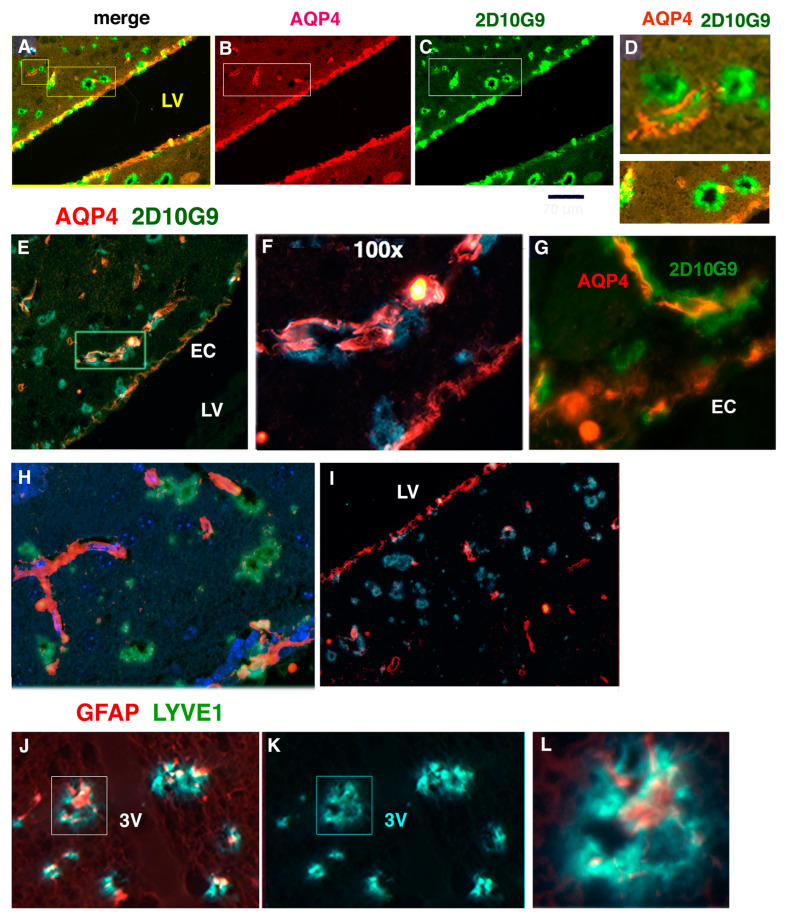
Colocalization of AQP4 and HOCl-modifed epitopes in vessels near the lateral ventricles suggest a role for MPO in glymphatic impairment (**A**–**C**). Immunostaining of hMPO-A53T brain shows 2D10G9 and AQP4 costained in a subset of ependymal cells lining the lateral ventricle (LV) (**A**). Scale bar under C, 70 um. (**B**) AQP4 staining is seen in the ependymal cells and a few vessels in the boxed area. (**C**) 2D10G9 staining is seen in adjacent vessels in the boxed area and in some ependymal cells. (**D**) The small box in Panel (**A**) is enlarged in (**D**) to show two 2D10G9 positive vessels (green) with AQP4 (red) in filaments or astrocytic sheath encircling the vessel. The lower Panel shows a region from the big box in A with 2D10G9 stained vessels (green) colocalizing with AQP4 (red). (**E**–**I**) Panel (**E**) shows AQP4 (red) and 2D10G9 (green) staining along a vessel near the ependymal cells (EC) of the lateral ventricle (LV). The boxed area is enlarged in panel (**F**) showing what appears to be distinct layers of AQP4 and 2D10G9 staining. Panel (**G**) shows another example of AQP4 and 2D10G9 in layered staining along a vessel. Panel (**H**) show AQP4 (red) in vessels whose ends interact with 2D10G9 (green) stained vessels. Panel (**I**) shows AQP4 again in vessels that contact 2D10G9 vessels without colocalizing. Panel (**J**) show colocalization of GFAP (red) astrocyte marker, and LYVE1 (green) glymphatic marker in the vessels along the 3rd ventricle (3V) (not DAPI stained). Panel (**K**) shows LYVE1 staining only, which allows visualization of lumens in some vessels. Panel (**L**) shows the boxed area in Panel (**J**) enlarged to reveal lumens in LYVE1+ vessels around GFAP astrocyte marker (red).20× and 40× objectives was used in (**A**–**E**), (**H**–**K**), and 100× objective in (**F**,**G**,**L**).

AQP4 is a water channel protein present in astrocytic end feet which form a protective sheath around vessels, creating a peri-vascular space in which CSF can flow into the brain vasculature, then exit through AQP4 channels into astrocytes and exiting to the parenchyma providing water, CSF, and nutrients. In Figure 6, we see interaction between AQP4 and HOCl-modified epitopes on dystrophic glymphatic vessels near lateral ventricles. MPO oxidants are likely to damage the glymphatic vessels in the hMPO-A53T model, potentially impairing the glymphatic waste removal system, contributing to the observed motor impairment.

In hMPO-A53T brain, AQP4 (red) (Figure 6B) is detected in ependymal cells lining the lateral ventricles and in some nearby vessels. Immunostaining for HOCl-modified epitopes using 2D10G9 is observed in some of the ependymal cells and nearby vessels (Figure 6B–D merged in Figure 6A). Traces of AQP4 staining are associated with these 2D10G9 positive vessels (boxed area in Figure 6B,C, enlarged in Figure 6D). The small box in Figure 6, Panel A, is enlarged in Panel D to show two green 2D10G9 stained vessels partly encircled with red AQP4 filaments, possibly astrocytic end feet wrapping glymphatic vessels. The lower box in Figure 6, Panel D shows another example of AQP4 (red) association with HOCl modified epitopes (green) in vessels at the ependymal layer.

In another example, AQP4 (red) (Figure 6E, box enlarged in Figure 6F) is associated with HOCl-modified epitopes (green) along a vessel. Another example is shown in Figure 6, Panel G with AQP4 (red) aligned with 2D10G9 stained vessels. Figure 6, Panels H and I show several 2D10G9 positive stained vessels (green) contacting AQP4 positive vessels (red) near the ependymal layer.

As further evidence that the AQP4 detected in vessels along the ventricles is in astrocytic end feet, Figure 6 (Panels J, K, and L) shows vessels along the third ventricle that stain for LYVE1, a marker for glymphatic vessels (green) and GFAP (red), a marker for astrocytes. At higher magnification (Figure 6L), there appears to be several openings in LYVE1-positive vessels associated with GFAP-positive astrocytes. The association of HOCl-modified epitopes with glymphatic vessels could be linked to impaired clearance of waste products including oxidized αSyn from hMPO-A53T brain.

## 4. Discussion

### 4.1. Deleterious Versus Protective Effects of MPO

PD is a major neurodegenerative disease with only limited therapeutic options. Previously we have shown that MPO oxidation products are a major contributor to motor deficits and oxidative damage in the mouse model of PD, hMPO-A53T [31]. In the current study we found that supplying a less toxic, alternative substrate to MPO, namely SCN^−^, we could reduce the level of the more toxic HOCl-modified epitopes and improve motor behavior in the hMPO-A53T mouse model. In an unexpected result we also demonstrated in this mouse model that there was significant oxidative damage in vessels we determined were glymphatic vessels along the lateral, 3rd and 4th ventricles. This oxidative damage was reduced when the mice were treated with SCN^−^ in keeping with our hypothesis that providing a less toxic substrate to MPO will reduce damage to the surrounding tissues. Importantly, this further suggests that MPO oxidation of glymphatic vessels impedes clearance of oxidized αSyn and other waste, thereby exacerbating PD.

The MPO-H_2_O_2_ system catalyzes halogenation of SCN^−^ to form hypothiocyanous acid (HOSCN) [63]. HOSCN plays a beneficial role as a bacteriostatic agent but may also have deleterious effects on normal cellular function [64]. MPO-catalyzed oxidation of SCN^−^ has been linked to carbamylation of lipoproteins such as LDL [65] and lipoproteins of the high density range [66], leading to pro-atherosclerotic events including cholesterol accumulation and foam-cell formation (reviewed in Ref. [67]). Sources of SCN^−^ include tobacco smoke, environmental pollutants, diet, or the metabolism of cyanide by sulfurtransferases [68]. Recently, an alternative source of cyanate has been identified [69]. MPO was found to catalyze the two-electron oxidation of cyanide to cyanate and promote the carbamylation of taurine, lysine and LDL [69].

SCN^−^ may have beneficial effects therapeutically due to the fact that HOSCN is less toxic than HOCl [70,71]. HOSCN is a less powerful oxidizing agent compared to HOCl and HOBr [72,73]. In a recent study using murine macrophages, HOSCN was shown to modulate damage caused by HOCl [46,74]. Several animal studies have been performed suggesting that SCN^−^ treatment may be therapeutically useful. Nebulized SCN^−^ effectively reduced bacterial load, infection mediated morbidity, and airway inflammation in mice infected with *P*. *aeruginosa* [75]. In a cystic fibrosis mouse model nebulized SCN^−^ was administered to mice and was shown to significantly decrease airway neutrophil infiltrate and restore the redox ratio of glutathione in lung tissue and airway epithelial lining fluid to levels comparable to WT [71]. 

In another example, supplementation of SCN^−^ in the drinking water was tested as a possible therapy to reduce the oxidative damage by HOCl. When humanized MPO mice were crossed to LDLR^−/−^ mice and treated with or without SCN^−^ in the drinking water, there was a 2-fold increase in plasma SCN^−^ and a 26% reduction in plaque area in the SCN^−^ treated mice compared to control mice [49]. In another mouse model of atherosclerosis, SCN^−^ supplementation also reduced plaque size in apolipoprotein E^−/−^ mice and reduced oxidative damage while improving endothelial function [76]. In a rat model of myocardial ischemia-reperfusion injury oral dosing of rats with SCN^−^ before acute ischemia-reperfusion injury significantly reduced the infarct size as a percentage of the total reperfused area (54% versus 74%) and increased the salvageable area (46% versus 26%) as determined by MRI imaging [77]. Further studies are warranted to evaluate the anti-inflammatory effects of SCN^−^ in chronic neurodegenerative diseases involving MPO such as PD, in that SCN^−^ may counter the negative impact of MPO/HOCl and MPO/nitration.

We cannot rule out other pathways to reduce the hMPO injury response with SCN^−^ treatment namely, the contribution of the redox system, (e.g., thioredoxin reductase) which may also play a role in reducing oxidative damage [78,79]. It has been reported that HOSCN formation could have detrimental effects on the basis of alterations in redox signaling (e.g., targeting free cysteines from low-molecular mass protein residues) and promotion of cellular dysfunction following HOSCN treatment of cellular models [78,79]. The formation of reversible oxidation products from HOSCN and its catalytic reduction via direct reaction with mammalian thioreductase via electron transfer from NADPH and reduction of disulfides and other substrates could also contribute to the improvement we see in motor behavior [78,80].

### 4.2. Impairment of the Glymphatic Waste Clearance System in hMPO-A53T Mice

The glial-lymphatic or glymphatic system is a fluid transport mechanism that delivers CSF and nutrients into the brain along peri-arterial channels created by interlocking astrocytic end feet that wrap around the vessels creating a peri-arterial space for CSF to flow into the brain [81,82]. This system utilizes the AQP4 water channel protein which is highly localized in orthogonal arrays on the astrocyte end feet, allowing transport of water and nutrients into astrocytes and then into the interstitial space of the brain. Waste products such as oxidized αSyn are propelled by convective flux produced by heart contractions through the interstitial spaces and finally into peri-venous channels that lead out of the brain.

Our findings raise the possibility that MPO-mediated oxidation of αSyn in the hMPO-A53T model creates insoluble misfolded protein aggregates that adhere to the walls of the glymphatic vessels, causing stiffening of the walls such that the vessels cannot deflate and inflate with the convective flux of CSF into the perivascular spaces. This could explain the enlarged peri-ventricular vessels around the LVs in the hMPO-A53T brain as compared to smaller and flatter vessels in A53T mouse brain, or the lack of peri-ventricular vessels in WT or transgenic hMPO brain. Deposition of these oxidized or aggregated proteins (e.g., hMPO, nitSyn, 2D10G9 HOCl oxidized epitopes, carbsyn, LYVE1, GFAP, AQP4) may prevent the collapse of the glymphatic vessel walls. Glymphatic vessels were for some years undetectable in normal mouse brains because these vessels collapse at the time of death or fixation [83]. Only in the presence of hMPO and A53T αSyn expression do we significantly detect dystrophic vessels coated by MPO, αSyn, AQP4, and GFAP, presumably because these deposits prevent the collapse of the vessel walls. Such deposits may also block valves in lymphatic vessels that normally allow outflow of CSF and nutrients into the brain parenchyma.

Recently, the glymphatic system has become a research focus due to its involvement in neurodegenerative diseases, notably AD and PD. While the original credit for a waste disposal system belongs to B. Lewis [84] and H. Obersteiner [85], the concept has been recently rediscovered [82] (reviewed in Ref. [86]). Soon after this discovery the meningial glymphatic system was uncovered [87]. A more recent study used IHC to define the position of glymphatic markers in 12 areas of human brain and together these reports have helped to generate a promising field of research [88]. There is however continued debate as to the mechanisms of flow in the glymphatic system as well as the role of the glymphatic system in normal homeostasis and neurodegenerative diseases [89,90,91,92,93].

### 4.3. The Possible Role of MPO in Glymphatics in PD

The work presented here suggests an active role for MPO in the impairment of the glymphatic system in the vessels surrounding the lateral, 3rd, and 4th ventricles in the hMPO-A53T mouse model of PD. These glymphatic vessels were immunostained for MPO and for HOCl modified epitopes. Since MPO is the only peroxidase able to generate HOCl, this result indicates that MPO is enzymatically active and able to oxidize proteins and probably also lipids (via intermediate formation of HOCl) in these vessels. These vessels were also stained with the glymphatic marker LYVE1. Furthermore, in the same vessels there is immunostaining for GFAP, a marker for activated astrocytes. Finally, the astrocytes in these vessels were immunostained for AQP4, a water channel that intakes water from the peri-arterial vessels and transports it through astrocytes and interstitial fluid to wash out waste such as αSyn from the brain. These results point to a role for MPO in the impairment of the glymphatic system leading to development of PD in this murine model system. Interestingly, there was no MPO immunostaining in periventricular vessels in the hMPO transgenic mice lacking A53T, nor in WT mice. Thus, it is the genetic background of the hMPO mice crossed to the A53T mice that gives rise to MPO expression and formation of HOCl-modified epitopes, leading to deposition of oxidized proteins on the glymphatic walls, impairing transport of oxidized waste out of the brain parenchyma.

One interesting finding is that the ependymal cells that line the ventricles are often killed by close proximity of the peri-ventricular glymphatic vessels, suggesting that MPO oxidants or nitrated/chlorinated αSyn are toxic to these ependymal cells. This would likely contribute to the greater motor disability of the MPO αSyn mice due to loss of integrity of the blood–brain barrier.

It is hypothesized that failures of the glymphatic system to successfully remove misfolded and oxidized proteins contributes to PD. The question remains as to where the MPO protein in these vessels originates. First, the MPO gene could be activated in astrocytes that encase the vessels to create the peri-arterial channel. We previously showed that the hMPO transgene is expressed at high levels in astrocytes around amyloid β plaques in the hMPO-APP23 model [27], and here we show MPO is expressed in subsets of astrocytes in hMPO-A53T (Figure 3U,V and Figure 5A). We further have shown that in the hMPO-A53T model, this peroxidase can be expressed in neurons [31]. The MPO expressed there is a 90kD unprocessed form that is secreted. This would fit the current results as secreted 90kD MPO could be enzymatically active in and around the vessels which would explain the oxidative damage at the vessels. Several years-ago experiments were conducted that support this hypothesis. Horseradish peroxidase was infused into the lateral cerebral ventricles of anesthetized cats and dogs [94]. The flow of the peroxidase then proceeded towards the perivenous space [94]. This may be relevant to the current studies since horseradish peroxidase and MPO are functionally related it may be that hMPO expressed in the brains of our mouse PD model follows a similar path. The results presented here show that there is significant oxidation in the vessels around the ventricles which most likely prevents their collapse during fixation. We cannot rule out the possibility that MPO is transported into the parenchyma by unknown transporters [81] or by extracellular vesicles [95]. Interestingly, we have recently shown that MPO can be encapsulated by extracellular vesicles and is enzymatically active (unpublished observation). It will be particularly interesting to follow up the analysis of this model system using a variety of techniques to follow glymphatic flow under different conditions.

The presence of MPO along with HOCl-modified epitopes in the vessels argues that MPO is enzymatically active and further suggests that oxidation of αSyn as well as other proteins is occurring. It is therefore not surprising to see deficits in motor ability in the hMPO-A53T model system. A recent report identified MPO in the CSF of PD [96]. While the authors of this study did not find a correlation between CSF MPO in PD patients versus healthy controls, they did notice a correlation of MPO concentration with disease duration [96]. We and others have shown that MPO expression is implicated in PD [29,31,97,98,99,100]. Furthermore, we and others have also shown that there is an increase in MPO in neurons and activated glial cells in PD [29,31,101,102,103]. As mentioned above, normally MPO is only expressed in myeloid cells. The abnormal expression in neurons and glial cells in neurodegenerative diseases suggests that MPO can promote these diseases.

These findings suggest that MPO may be a good target for the development of therapeutics to block its activity (reviewed in Ref. [104]). The results presented here provide evidence that SCN^−^ treatment can improve activities in several motor tests. While SCN^−^ may not be a viable therapeutic itself for the treatment of PD due to toxicity issues, several other studies have hinted that MPO inhibitors can have a beneficial effect for the treatment of neurodegenerative diseases in humans. A phase I study using an MPO inhibitor to treat PD was recently completed [105]. Studies in animal models of Multiple Sclerosis (EAE) [106], multiple system atrophy [107,108] and in an animal model of amyotrophic lateral sclerosis [109] indicate that blocking MPO activity lessens disease and improves motor behavior, pointing to the usefulness of developing MPO inhibitors. A safe and therapeutically effective blocker of MPO may be beneficial in other diseases where there is abnormal stress inducing MPO expression and causing oxidative damage. Side effects from blockage of MPO are not to be expected, since MPO deficient individuals are mostly asymptomatic.

## Data Availability

The data is contained within the article.

## Abbreviations

αSyn	Alpha synuclein
AD	Alzheimer’s disease
AQP4	aquaporin 4
Br^−^	bromide
Cl^−^	chloride
CSF	cerebral spinal fluid
DAPI	4′, 6-diamidino-2-phenylindole
GFAP	glial fibrillary acidic protein
hMPO	human MPO
HOBr	hypobromous acid
HOCl	hypochlorous acid
HOI	hypoiodous acid
HOSCN	hypothiocyanous acid
I^−^	iodide
IHC	immunohistochemistry
LV	lateral ventricle
LYVE1	lymphatic vessel endothelial hyaluronan receptor 1
mAb	monoclonal antibody
mMPO	murine MPO
MPO	myeloperoxidase
NaSCN	sodium thiocyanate
pAb	polyclonal antibody
PBS	phosphate-buffered saline
PBST	PBS + 0.05% Tween 20
PD	Parkinson’s disease
ROS	reactive oxygen species
SCN^−^	thiocyanate
Thy1	human thymus cell antigen 1

## References

[1] Goetz C.G., McGhiey A. (2011). The movement disorder society and movement disorders: A modern history. Mov. Disord..

[2] Poewe W., Seppi K., Tanner C.M., Halliday G.M., Brundin P., Volkmann J., Schrag A.E., Lang A.E. (2017). Parkinson disease. Nat. Rev. Dis. Prim..

[3] Chavarria C., Souza J.M. (2013). Oxidation and nitration of alpha-synuclein and their implications in neurodegenerative diseases. Arch. Biochem. Biophys..

[4] He Y.X., Yu Z.W., Chen S.D. (2018). Alpha-synuclein nitration and its implications in Parkinson’s disease. ACS Chem. Neurosci..

[5] Perry T.L., Yong V.W. (1986). Idiopathic Parkinson’s disease, progressive supranuclear palsy and glutathione metabolism in the substantia nigra of patients. Neurosci. Lett..

[6] Jenner P., Dexter D.T., Sian J., Schapira A.H., Marsden C.D. (1992). Oxidative stress as a cause of nigral cell death in Parkinson’s disease and incidental Lewy body disease. The Royal Kings and Queens Parkinson’s Disease Research Group. Ann. Neurol..

[7] Yoritaka A., Hattori N., Uchida K., Tanaka M., Stadtman E.R., Mizuno Y. (1996). Immunohistochemical detection of 4-hydroxynonenal protein adducts in Parkinson disease. Proc. Natl. Acad. Sci. USA.

[8] Duda J.E., Giasson B.I., Mabon M.E., Lee V.M., Trojanowski J.Q. (2002). Novel antibodies to synuclein show abundant striatal pathology in Lewy body diseases. Ann. Neurol..

[9] Nunomura A., Moreira P.I., Lee H.G., Zhu X., Castellani R.J., Smith M.A., Perry G. (2007). Neuronal death and survival under oxidative stress in Alzheimer and Parkinson diseases. CNS Neurol. Disord. Drug Targets.

[10] Percario S., Barbosa A.d., Varela E.L., Gomes A.R.Q., Ferreira M.E.S., Moreira T.d.A., Dolabela M.F. (2020). Oxidative Stress in Parkinson’s Disease: Potential Benefits of Antioxidant Supplementation. Oxid. Med. Cell Longev..

[11] Malle E., Buch T., Grone H.J. (2003). Myeloperoxidase in kidney disease. Kidney Int..

[12] Arnhold J., Malle E. (2022). Halogenation Activity of Mammalian Heme Peroxidases. Antioxidants.

[13] Klebanoff S.J., Kettle A.J., Rosen H., Winterbourn C.C., Nauseef W.M. (2013). Myeloperoxidase: A front-line defender against phagocytosed microorganisms. J. Leukoc Biol..

[14] Winterbourn C.C., Kettle A.J., Hampton M.B. (2016). Reactive Oxygen Species and Neutrophil Function. Ann. Rev. Biochem..

[15] Hawkins C.L., Davies M.J. (2021). Role of myeloperoxidase and oxidant formation in the extracellular environment in inflammation-induced tissue damage. Free Radic. Biol. Med..

[16] Davies M.J. (2021). Myeloperoxidase: Mechanisms, reactions and inhibition as a therapeutic strategy in inflammatory diseases. Pharmacol. Ther..

[17] Xu S., Chuang C.Y., Malle E., Gamon L.F., Hawkins C.L., Davies M.J. (2022). Influence of plasma halide, pseudohalide and nitrite ions on myeloperoxidase-mediated protein and extracellular matrix damage. Free Radic. Biol. Med..

[18] Siraki A.G. (2021). The many roles of myeloperoxidase: From inflammation and immunity to biomarkers, drug metabolism and drug discovery. Redox. Biol..

[19] Davies M.J., Hawkins C.L. (2020). The Role of Myeloperoxidase in Biomolecule Modification, Chronic Inflammation, and Disease. Antioxid. Redox. Signal.

[20] Hoy A., Tregouet D., Leininger-Muller B., Poirier O., Maurice M., Sass C., Siest G., Tiret L., Visvikis S. (2001). Serum myeloperoxidase concentration in a healthy population: Biological variations, familial resemblance and new genetic polymorphisms. Eur. J. Hum. Genet.

[21] Pecoits-Filho R., Stenvinkel P., Marchlewska A., Heimburger O., Bárány P., Hoff C.M., Holmes C.J., Suliman M., Lindholm B., Schalling M. (2003). A functional variant of the myeloperoxidase gene is associated with cardiovascular disease in end-stage renal disease patients. Kidney Int. Suppl.

[22] Makela R., Karhunen P.J., Kunnas T.A., Ilveskoski E., Kajander O.A., Mikkelsson J., Perola M., Penttila A., Lehtimaki T. (2003). Myeloperoxidase gene variation as a determinant of atherosclerosis progression in the abdominal and thoracic aorta: An autopsy study. Lab. Invest..

[23] Makela R., Laaksonen R., Janatuinen T., Vesalainen R., Nuutila P., Jaakkola O., Knuuti J., Lehtimaki T. (2004). Myeloperoxidase gene variation and coronary flow reserve in young healthy men. J. Biomed. Sci..

[24] Asselbergs F.W., Reynolds W.F., Cohen-Tervaert J.W., Jessurun G.A., Tio R.A. (2004). Myeloperoxidase polymorphism related to cardiovascular events in coronary artery disease. Am. J. Med..

[25] Rudolph V., Rudolph T.K., Kubala L., Clauberg N., Maas R., Pekarova M., Klinke A., Lau D., Szöcs K., Meinertz T. (2009). A myeloperoxidase promoter polymorphism is independently associated with mortality in patients with impaired left ventricular function. Free Radic. Biol. Med..

[26] Reynolds W.F., Rhees J., Maciejewski D., Paladino T., Sieburg H., Maki R.A., Masliah E. (1999). Myeloperoxidase polymorphism is associated with gender specific risk for Alzheimer’s disease. Exp. Neurol..

[27] Maki R.A., Tyurin V.A., Lyon R.C., Hamilton R.L., DeKosky S.T., Kagan V.E., Reynolds W.F. (2009). Aberrant expression of myeloperoxidase in astrocytes promotes phospholipid oxidation and memory deficits in a mouse model of Alzheimer disease. J. Biol. Chem..

[28] Green P.S., Mendez A.J., Jacob J.S., Crowley J.R., Growdon W., Hyman B.T., Heinecke J.W. (2004). Neuronal expression of myeloperoxidase is increased in Alzheimer’s disease. J. Neurochem..

[29] Choi D.-K., Pennathur S., Perier C., Tieu K., Teismann P., Wu D.-C., Jackson-Lewis V., Vila M., Vonsattel J.-P., Heinecke J.W. (2005). Ablation of the inflammatory enzyme myeloperoxidase mitigates features of Parkinson’s disease in mice. J. Neurosci..

[30] Gellhaar S., Sunnemark D., Eriksson H., Olson L., Galter D. (2017). Myeloperoxidase-immunoreactive cells are significantly increased in brain areas affected by neurodegeneration in Parkinson’s and Alzheimer’s disease. Cell Tissue Res..

[31] Maki R.A., Holzer M., Motamedchaboki K., Malle E., Masliah E., Marsche G., Reynolds W.F. (2019). Human myeloperoxidase (hMPO) is expressed in neurons in the substantia nigra in Parkinson’s disease and in the hMPO-alpha-synuclein-A53T mouse model, correlating with increased nitration and aggregation of alpha-synuclein and exacerbation of motor impairment. Free Radic. Biol. Med..

[32] Reynolds W.F., Maki R.A., Hawkins C., Nauseef W.M. (2021). The role of myeloperoxidase in neurodegenerative disease. Mammalian Heme Peroxidases: Diverse Roles in Health and Disease.

[33] Castellani L.W., Chang J.J., Wang X., Lusis A.J., Reynolds W.F. (2006). Transgenic mice express human MPO -463G/A alleles at atherosclerotic lesions, developing hyperlipidemia and obesity in -463G males. J. Lipid. Res..

[34] Kumar A.P., Piedrafita F.J., Reynolds W.F. (2004). Peroxisome proliferator-activated receptor gamma ligands regulate myeloperoxidase expression in macrophages by an estrogen-dependent mechanism involving the -463GA promoter polymorphism. J. Biol. Chem..

[35] Piedrafita F.J., Molander R.B., Vansant G., Orlova E.A., Pfahl M., Reynolds W.F. (1996). An Alu element in the myeloperoxidase promoter contains a composite SP1-thyroid hormone-retinoic acid response element. J. Biol. Chem..

[36] Reynolds W.F., Kumar A., Piedrafita F.J. (2006). The human myeloperoxidase gene is regulated by LXR and PPARalpha ligands. Biochem. Biophys. Res. Commun..

[37] Vansant G., Reynolds W.F. (1995). The consensus sequence of a major Alu subfamily contains a functional retinoic acid response element. Proc. Natl. Acad. Sci. USA.

[38] Reynolds W.F., Hiltunen M., Pirskanen M., Mannermaa A., Helisalmi S., Lehtovirta M., Alafuzoff I., Soininen H. (2000). MPO and APOEepsilon4 polymorphisms interact to increase risk for AD in Finnish males. Neurology.

[39] Crawford F.C., Freeman M.J., Schinka J.A., Morris M.D., Abdullah L.I., Richards D., Sevush S., Duara R., Mullan M.J. (2001). Association between Alzheimer’s disease and a functional polymorphism in the Myeloperoxidase gene. Exp. Neurol..

[40] Leininger-Muller B., Hoy A., Herbeth B., Pfister M., Serot J.M., Stavljenic-Rukavina M., Massana L., Passmore P., Siest G., Visvikis S. (2003). Myeloperoxidase G-463A polymorphism and Alzheimer’s disease in the ApoEurope study. Neurosci. Lett..

[41] Zappia M., Manna I., Serra P., Cittadella R., Andreoli V., La Russa A., Annesi F., Spadafora P., Romeo N., Nicoletti G. (2004). Increased risk for Alzheimer disease with the interaction of MPO and A2M polymorphisms. Arch. Neurol..

[42] Pope S.K., Kritchevsky S.B., Ambrosone C., Yaffe K., Tylavsky F., Simonsick E.M., Rosano C., Stewart S., Harris T. (2006). Myeloperoxidase polymorphism and cognitive decline in older adults in the Health, Aging, and Body Composition Study. Am. J. Epidemiol..

[43] Schabath M.B., Spitz M.R., Hong W.K., Delclos G.L., Reynolds W.F., Gunn G.B., Whitehead L.W., Wu X. (2002). A myeloperoxidase polymorphism associated with reduced risk of lung cancer. Lung Cancer.

[44] Yang J.-P., Wang W.-B., Yang X.-X., Yang L., Ren L., Zhou F.-X., Hu L., He W., Li B.-Y., Zhu Y. (2013). The MPO-463G>A polymorphism and lung cancer risk: A meta-analysis based on 22 case-control studies. PLoS ONE.

[45] Castillo-Tong D.C., Pils D., Heinze G., Braicu I., Sehouli J., Reinthaller A., Schuster E., Wolf A., Watrowski R., Maki R.A. (2014). Association of myeloperoxidase with ovarian cancer. Tumour Biol..

[46] Guo C., Davies M.J., Hawkins C.L. (2020). Role of thiocyanate in the modulation of myeloperoxidase-derived oxidant induced damage to macrophages. Redox Biol..

[47] Vanichkitrungruang S., Chuang C.Y., Hawkins C.L., Hammer A., Hoefler G., Malle E., Davies M.J. (2019). Oxidation of human plasma fibronectin by inflammatory oxidants perturbs endothelial cell function. Free Radic. Biol. Med..

[48] Flouda K., Gammelgaard B., Davies M.J., Hawkins C.L. (2021). Modulation of hypochlorous acid (HOCl) induced damage to vascular smooth muscle cells by thiocyanate and selenium analogues. Redox Biol..

[49] Morgan P.E., Laura R., Maki R.A., Reynolds W.F., Davies M.J. (2015). Thiocyanate supplementation decreases atherosclerotic plaque in mice expressing human myeloperoxidase. Free Radic. Res..

[50] Hablitz L.M., Nedergaard M. (2021). The glymphatic system. Curr. Biol..

[51] Rasmussen M.K., Mestre H., Nedergaard M. (2022). Fluid transport in the brain. Physiol. Rev..

[52] Laura R.P., Dong D., Reynolds W.F., Maki R.A. (2016). T47D Cells Expressing Myeloperoxidase Are Able to Process, Traffic and Store the Mature Protein in Lysosomes: Studies in T47D Cells Reveal a Role for Cys319 in MPO Biosynthesis that Precedes Its Known Role in Inter-Molecular Disulfide Bond Formation. PLoS ONE.

[53] Baba M., Nakajo S., Tu P.H., Tomita T., Nakaya K., Lee V.M., Trojanowski J.Q., Iwatsubo T. (1998). Aggregation of alpha-synuclein in Lewy bodies of sporadic Parkinson’s disease and dementia with Lewy bodies. Am. J. Pathol..

[54] Giasson B.I., Duda J.E., Murray I.V., Chen Q., Souza J.M., Hurtig H.I., Ischiropoulos H., Trojanowski J.Q., Lee V.M. (2000). Oxidative damage linked to neurodegeneration by selective alpha-synuclein nitration in synucleinopathy lesions. Science.

[55] Malle E., Woenckhaus C., Waeg G., Esterbauer H., Grone E.F., Grone H.J. (1997). Immunological evidence for hypochlorite-modified proteins in human kidney. Am. J. Pathol..

[56] Malle E., Hazell L., Stocker R., Sattler W., Esterbauer H., Waeg G. (1995). Immunologic detection and measurement of hypochlorite-modified LDL with specific monoclonal antibodies. Arter. Thromb. Vasc. Biol..

[57] Masliah E., Rockenstein E., Veinbergs I., Mallory M., Hashimoto M., Takeda A., Sagara Y., Sisk A., Mucke L. (2000). Dopaminergic loss and inclusion body formation in alpha-synuclein mice: Implications for neurodegenerative disorders. Science.

[58] Games D., Seubert P., Rockenstein E., Patrick C., Trejo M., Ubhi K., Ettle B., Ghassemiam M., Barbour R., Schenk D. (2013). Axonopathy in an alpha-synuclein transgenic model of Lewy body disease is associated with extensive accumulation of C-terminal-truncated alpha-synuclein. Am. J. Pathol..

[59] Kumar A.P., Ryan C., Cordy V., Reynolds W.F. (2005). Inducible nitric oxide synthase expression is inhibited by myeloperoxidase. Nitric Oxide.

[60] Chandra S., Gallardo G., Fernandez-Chacon R., Schluter O.M., Sudhof T.C. (2005). Alpha-synuclein cooperates with CSPalpha in preventing neurodegeneration. Cell.

[61] Martin L.J., Semenkow S., Hanaford A., Wong M. (2014). Mitochondrial permeability transition pore regulates Parkinson’s disease development in mutant alpha-synuclein transgenic mice. Neurobiol. Aging.

[62] Rothman S.M., Griffioen K.J., Vranis N., Ladenheim B., Cong W.N., Cadet J.L., Haran J., Martin B., Mattson M. (2013). Neuronal expression of familial Parkinson’s disease A53T alpha-synuclein causes early motor impairment, reduced anxiety and potential sleep disturbances in mice. J. Park. Dis..

[63] van Dalen C.J., Whitehouse M.W., Winterbourn C.C., Kettle A.J. (1997). Thiocyanate and chloride as competing substrates for myeloperoxidase. Biochem. J..

[64] Delanghe S., Delanghe J.R., Speeckaert R., Van Biesen W., Speeckaert M.M. (2017). Mechanisms and consequences of carbamoylation. Nat. Rev. Nephrol..

[65] Wang Z., Nicholls S., Rodriguez E.R., Kummu O., Hörkkö S., Barnard J.W., Reynolds W.F., Topol E., A DiDonato J., Hazen S.L. (2007). Protein carbamylation links inflammation, smoking, uremia and atherogenesis. Nat. Med..

[66] Holzer M., Gauster M., Pfeifer T., Wadsack C., Fauler G., Stiegler P., Koefeler H., Beubler E., Schuligoi R., Heinemann A. (2011). Protein carbamylation renders high-density lipoprotein dysfunctional. Antioxid. Redox. Signal.

[67] Marsche G., Stadler J.T., Kargl J., Holzer M. (2022). Understanding Myeloperoxidase-Induced Damage to HDL Structure and Function in the Vessel Wall: Implications for HDL-Based Therapies. Antioxidants.

[68] Nagahara N., Okazaki T., Nishino T. (1995). Cytosolic mercaptopyruvate sulfurtransferase is evolutionarily related to mitochondrial rhodanese. Striking similarity in active site amino acid sequence and the increase in the mercaptopyruvate sulfurtransferase activity of rhodanese by site-directed mutagenesis. J. Biol. Chem..

[69] Delporte C., Boudjeltia K.Z., Furtmueller P.G., Maki R.A., Dieu M., Noyon C., Soudi M., Dufour D., Coremans C., Nuyens V. (2018). Myeloperoxidase-catalyzed oxidation of cyanide to cyanate: A potential carbamylation route involved in the formation of atherosclerotic plaques?. J. Biol. Chem..

[70] Wagner B.A., Reszka K.J., McCormick M.L., Britigan B.E., Evig C.B., Burns C. (2004). Role of thiocyanate, bromide and hypobromous acid in hydrogen peroxide-induced apoptosis. Free Radic. Res..

[71] Chandler J.D., Day B.J. (2015). Biochemical mechanisms and therapeutic potential of pseudohalide thiocyanate in human health. Free Radic Res..

[72] Skaff O., Pattison D.I., Davies M.J. (2009). Hypothiocyanous acid reactivity with low-molecular-mass and protein thiols: Absolute rate constants and assessment of biological relevance. Biochem. J..

[73] Talib J., Pattison D.I., Harmer J.A., Celermajer D.S., Davies M.J. (2012). High plasma thiocyanate levels modulate protein damage induced by myeloperoxidase and perturb measurement of 3-chlorotyrosine. Free Radic. Biol. Med..

[74] Guo C., Sileikaite I., Davies M.J., Hawkins C.L. (2020). Myeloperoxidase Modulates Hydrogen Peroxide Mediated Cellular Damage in Murine Macrophages. Antioxidants.

[75] Chandler J.D., Min E., Huang J., Nichols D., Day B.J. (2013). Nebulized thiocyanate improves lung infection outcomes in mice. Br. J. Pharmacol..

[76] Zietzer A., Niepmann S.T., Camara B., Lenart M.A., Jansen F., Becher M.U., Andrie R., Nickenig G., Tiyerili V. (2019). Sodium thiocyanate treatment attenuates atherosclerotic plaque formation and improves endothelial regeneration in mice. PLoS ONE.

[77] Hall L., Guo C., Tandy S., Broadhouse K., Dona A.C., Malle E., Bartels E.D., Christoffersen C., Grieve S.M., Figtree G. (2021). Oral pre-treatment with thiocyanate (SCN(-)) protects against myocardial ischaemia-reperfusion injury in rats. Sci. Rep..

[78] Love D.T., Guo C., Nikelshparg E.I., Brazhe N.A., Sosnovtseva O., Hawkins C.L. (2020). The role of the myeloperoxidase-derived oxidant hypothiocyanous acid (HOSCN) in the induction of mitochondrial dysfunction in macrophages. Redox Biol..

[79] Rayner B.S., Love D.T., Hawkins C.L. (2014). Comparative reactivity of myeloperoxidase-derived oxidants with mammalian cells. Free. Radic. Biol. Med..

[80] Day B.J. (2019). The science of licking your wounds: Function of oxidants in the innate immune system. Biochem. Pharmacol..

[81] Iliff J., Simon M. (2019). CrossTalk proposal: The glymphatic system supports convective exchange of cerebrospinal fluid and brain interstitial fluid that is mediated by perivascular aquaporin-4. J. Physiol..

[82] Iliff J.J., Wang M., Liao Y., Plogg B.A., Peng W., Gundersen G.A., Benveniste H., Vates G.E., Deane R., Goldman S.A. (2012). A paravascular pathway facilitates CSF flow through the brain parenchyma and the clearance of interstitial solutes, including amyloid beta. Sci. Transl. Med..

[83] Mestre H., Hablitz L.M., Xavier A.L.R., Feng W., Zou W., Pu T., Monai H., Murlidharan G., Rivera R.M.C., Simon M.J. (2018). Aquaporin-4-dependent glymphatic solute transport in the rodent brain. Elife.

[84] Lewis B. (2022). The relationships of the nerve-cell of the cortex to the llymphatic system of the brain. Proc. Roy. Soc. Lond..

[85] Obersteiner H. (1890). Introduction to the Study of the Anatomy of the Central Nervous Organs in Helath and Disease.

[86] Hablitz L.M., Nedergaard M. (2021). The Glymphatic System: A Novel Component of Fundamental Neurobiology. J. Neurosci..

[87] Louveau A., Smirnov I., Keyes T.J., Eccles J.D., Rouhani S.J., Peske J.D., Derecki N.C., Castle D., Mandell J.W., Lee K.S. (2015). Structural and functional features of central nervous system lymphatic vessels. Nature.

[88] Mezey E., Szalayova I., Hogden C.T., Brady A., Dosa A., Sotonyi P., Palkovits M. (2021). An immunohistochemical study of lymphatic elements in the human brain. Proc. Natl. Acad. Sci. USA.

[89] Pizzo M.E., Wolak D.J., Kumar N.N., Brunette E., Brunnquell C.L., Hannocks M., Abbott N.J., Meyerand M.E., Sorokin L., Stanimirovic D.B. (2018). Intrathecal antibody distribution in the rat brain: Surface diffusion, perivascular transport and osmotic enhancement of delivery. J. Physiol..

[90] Asgari M., de Zelicourt D., Kurtcuoglu V. (2016). Glymphatic solute transport does not require bulk flow. Sci. Rep..

[91] Smith A.J., Yao X., Dix J.A., Jin B.J., Verkman A.S. (2017). Test of the ‘glymphatic’ hypothesis demonstrates diffusive and aquaporin-4-independent solute transport in rodent brain parenchyma. Elife.

[92] Hladky S.B., Barrand M.A. (2014). Mechanisms of fluid movement into, through and out of the brain: Evaluation of the evidence. Fluids Barriers CNS.

[93] Proulx S.T. (2021). Cerebrospinal fluid outflow: A review of the historical and contemporary evidence for arachnoid villi, perineural routes, and dural lymphatics. Cell Mol. Life Sci..

[94] Rennels M.L., Gregory T.F., Blaumanis O.R., Fujimoto K., Grady P.A. (1985). Evidence for a ‘paravascular’ fluid circulation in the mammalian central nervous system, provided by the rapid distribution of tracer protein throughout the brain from the subarachnoid space. Brain Res..

[95] Balusu S., Van Wonterghem E., De Rycke R., Raemdonck K., Stremersch S., Gevaert K., Brkic M., Demeestere D., Vanhooren V., Hendrix A. (2016). Identification of a novel mechanism of blood-brain communication during peripheral inflammation via choroid plexus-derived extracellular vesicles. EMBO Mol. Med..

[96] Fernandez-Espejo E., de Fonseca F.R., Gavito A.L., Cordoba-Fernandez A., Chacon J., de Pablos A.M. (2022). Myeloperoxidase and Advanced Oxidation Protein Products in the Cerebrospinal Fluid in Women and Men with Parkinson’s Disease. Antioxidants.

[97] Teismann P. (2014). Myeloperoxidase in the neurodegenerative process of Parkinson’s disease. Dtsch. Med. Wochenschr..

[98] Yap Y.W., Whiteman M., Cheung N.S. (2007). Chlorinative stress: An under appreciated mediator of neurodegeneration?. Cell Signal.

[99] Ray R.S., Katyal A. (2016). Myeloperoxidase: Bridging the gap in neurodegeneration. Neurosci. Biobehav. Rev..

[100] Jeitner T.M., Kalogiannis M., Krasnikov B.F., Gomolin I., Peltier M.R., Moran G.R. (2016). Linking Inflammation and Parkinson Disease: Hypochlorous Acid Generates Parkinsonian Poisons. Toxicol. Sci..

[101] Chung Y.C., Kim S.R., Jin B.K. (2010). Paroxetine prevents loss of nigrostriatal dopaminergic neurons by inhibiting brain inflammation and oxidative stress in an experimental model of Parkinson’s disease. J. Immunol..

[102] Hirsch E.C., Hunot S. (2009). Neuroinflammation in Parkinson’s disease: A target for neuroprotection?. Lancet Neurol..

[103] Soubhye J., Aldib I., Delporte C., Prevost M., Dufrasne F., Antwerpen P.V. (2016). Myeloperoxidase as a Target for the Treatment of Inflammatory Syndromes: Mechanisms and Structure Activity Relationships of Inhibitors. Curr. Med. Chem..

[104] Kargapolova Y., Geissen S., Zheng R., Baldus S., Winkels H., Adam M. (2021). The Enzymatic and Non-Enzymatic Function of Myeloperoxidase (MPO) in Inflammatory Communication. Antioxidants.

[105] Jucaite A., Svenningsson P., Rinne J.O., Cselényi Z., Varnäs K., Johnström P., Amini N., Kirjavainen A.K., Helin S., Minkwitz M.C. (2015). Effect of the myeloperoxidase inhibitor AZD3241 on microglia: A PET study in Parkinson’s disease. Brain.

[106] Zhang H., Ray A., Miller N.M., Hartwig D., Pritchard K.A., Dittel B.N. (2016). Inhibition of myeloperoxidase at the peak of experimental autoimmune encephalomyelitis restores blood-brain barrier integrity and ameliorates disease severity. J. Neurochem..

[107] Stefanova N., Georgievska B., Eriksson H., Poewe W., Wenning G.K. (2012). Myeloperoxidase inhibition ameliorates multiple system atrophy-like degeneration in a transgenic mouse model. Neurotox Res..

[108] Kaindlstorfer C., Sommer P., Georgievska B., Mather R.J., Kugler A.R., Poewe W., Wenning G.K., Stefanova N. (2015). Failure of Neuroprotection Despite Microglial Suppression by Delayed-Start Myeloperoxidase Inhibition in a Model of Advanced Multiple System Atrophy: Clinical Implications. Neurotox Res..

[109] Peng J., Pan J., Mo J., Peng Y. (2022). MPO/HOCl Facilitates Apoptosis and Ferroptosis in the SOD1(G93A) Motor Neuron of Amyotrophic Lateral Sclerosis. Oxid Med. Cell Longev..

